# A Nomogram for Predicting Cancer‐Specific Survival in Young Patients With Advanced Lung Cancer Based on Competing Risk Model

**DOI:** 10.1111/crj.13800

**Published:** 2024-08-07

**Authors:** Jiaxin Li, Bolin Pan, Qiying Huang, Chulan Zhan, Tong Lin, Yangzhi Qiu, Honglang Zhang, Xiaohong Xie, Xinqin Lin, Ming Liu, Liqiang Wang, Chengzhi Zhou

**Affiliations:** ^1^ Department of Clinical Medicine Guangzhou Medical University Guangzhou China; ^2^ Department of Gastroenterology and Hepatology West China Hospital, Sichuan University China; ^3^ College of Life Science Henan University Kaifeng China; ^4^ Pulmonary and Critical Care Medicine, Guangzhou Institute of Respiratory Health, National Clinical Research Center for Respiratory Disease, National Center for Respiratory Medicine, State Key Laboratory of Respiratory Diseases The First Affiliated Hospital of Guangzhou Medical University Guangzhou Guangdong China

**Keywords:** competing risk model, nomogram, young lung cancer

## Abstract

**Background:**

Young lung cancer is a rare subgroup accounting for 5% of lung cancer. The aim of this study was to compare the causes of death (COD) among lung cancer patients of different age groups and construct a nomogram to predict cancer‐specific survival (CSS) in young patients with advanced stage.

**Methods:**

Lung cancer patients diagnosed between 2004 and 2015 were extracted from the Surveillance, Epidemiology, and End Results (SEER) database and stratified into the young (18–45 years) and old (> 45 years) groups to compare their COD. Young patients diagnosed with advanced stage (IVa and IVb) from 2010 to 2015 were reselected and divided into training and validation cohorts (7:3). Independent prognostic factors were identified through the Fine‐Gray's test and further integrated to the competing risk model. The area under the receiver operating characteristic curve (AUC), consistency index (C‐index), and calibration curve were applied for validation.

**Results:**

The proportion of cancer‐specific death (CSD) in young patients was higher than that in old patients with early‐stage lung cancer (*p* < 0.001), while there was no difference in the advanced stage (*p* = 0.999). Through univariate and multivariate analysis, 10 variables were identified as independent prognostic factors for CSS. The AUC of the 1‐, 3‐, and 5‐year prediction of CSS was 0.688, 0.706, and 0.791 in the training cohort and 0.747, 0.752, and 0.719 in the validation cohort. The calibration curves demonstrated great accuracy. The C‐index of the competing risk model was 0.692 (95% CI: 0.636–0.747) in the young patient cohort.

**Conclusion:**

Young lung cancer is a distinct entity with a different spectrum of competing risk events. The construction of our nomogram can provide new insights into the management of young patients with lung cancer.

## Graphical abstract

Young lung cancer is a distinct entity from elderly patients. We compare the cause of death between different age groups and identify the independent prognostic factors for young lung cancer through competing risk model. The prognosis of young lung cancer patients is significantly better than elderly patients. Miscellaneous malignant cancer was the common competing risk event of young lung cancer.

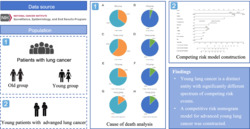

**Abbreviations**
95% CI: 95% confidence interval
AUC: the area under the receiver operating characteristic curve
CIF: cumulative incidence function
C‐index: consistency index
COD: causes of death
CS: Collaborative Stage
CSD: cancer‐specific death
CSS: cancer‐specific survival
LCSS: lung cancer‐specific survival
NSCLC: non–small cell lung cancer
OCD: other‐cause death
OS: overall survival
sdHR: subdistribution hazard ratio
SEER: Surveillance, Epidemiology, and End Results

## 1 Introduction

Lung cancer is more prevalent in the elderly population with high incidence and mortality, while young patients are considered a distinct entity [1, 2]. There is no unified definition of young lung cancer, and most studies currently define it as younger than 45 years old and older than 18 years old [3, 4]. As a rare subgroup accounting for 5% of lung cancers, young patients are more likely to be female, non‐White, nonsmokers, and adenocarcinoma [5, 6, 7]. Furthermore, young lung cancer has more aggressive tumor behavior with an advanced clinical stage at diagnosis, which may be related to insidious onset, atypical symptoms, difficulties in early diagnosis, and misdiagnosis [7, 8, 9].

Young lung cancer has better prognosis in median survival and overall survival (OS) than those in old patients due to more aggressive treatment intentions and better tolerability of combination therapies [9, 10]. In addition, the distinctive genetic characteristics observed in young patients suggest that they are often more amenable to targeted therapies, which is associated with favorable prognoses [11, 12]. Undeniably, multiple studies have indicated that young age is an independent prognostic protective factor for lung cancer [6, 13, 14, 15]. Moreover, in elderly patients with lung cancer, the presence of multiple comorbidities often leads to a significant increase in the probability of facing competing risk events with longer survival [16, 17, 18]. However, competing risk events in young lung cancer have not been thoroughly investigated yet.

Nomograms, which estimate the individual probability of recurrence or death based on independent prognostic factors, have become a widely used tool in clinical practice [19, 20]. Currently, a few studies have developed nomograms to predict OS and cancer‐specific survival (CSS) in young patients with non–small cell lung cancer (NSCLC), with limited consideration of competitive risk events or comprehensive evaluation of independent prognostic factors [21]. Considering the extremely poor prognosis, the management of advanced‐stage patients is often more challenging. However, unlike in elderly lung cancer, the analysis of competitive risk events and competitive risk assessment for advanced‐stage young patients remains deficient.

To explore the impact of different demographic characteristics and treatments on the prognosis of young lung cancer patients with advanced stage, we collected cases from the Surveillance, Epidemiology, and End Results (SEER) database between 2010 and 2015. Further, we constructed and validated a nomogram for young lung cancer patients by using competing risk model, hoping to provide an excellent reference for clinical decision‐making.

## 2 Methods

### 2.1 Data Source and Selection

**FIGURE 1:**
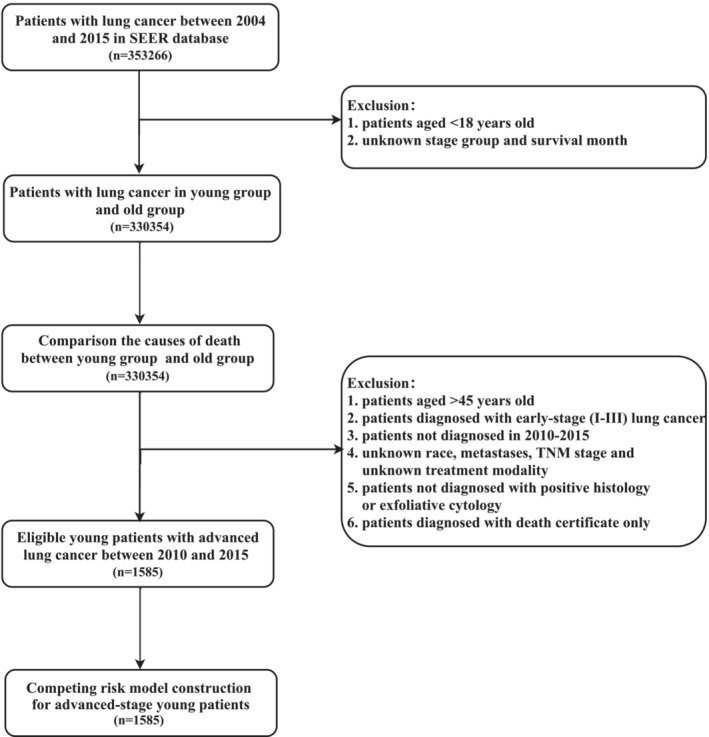
Flow diagram illustrating the screening process in the SEER database.

The SEER database contains information about the cancer incidence and survival of approximately 34.6% of the American population. Patients diagnosed with lung cancer were extracted from the SEER database. We reclassified the TNM stage based on Collaborative Stage (CS) information according to the AJCC eighth edition. A cohort comprising young and old patients was selected to compare the difference in death causes between different age groups. In the death cause analysis section, we kept as many samples with accurate causes of death to allow more accurate comparisons of their differences between different age groups. The inclusion criteria were as follows: (1) malignant tumor in lung and bronchus (primary sites: C34.0–34.9) from 2004 to 2015; (2) patients aged ≥ 18 and patients under 45 years old were considered as young lung cancer; and (3) lung cancer histology codes ranging from 8012 to 8576 of ICD‐O‐3. Subsequently, to construct the competing risk model for young lung cancer, we reselected patients aged 18–45 and diagnosed with advanced (IVa and IVb) lung cancer from 2010 to 2015 as the target cohort. This range was selected since SEER combined mets have been available since 2010. Additionally, patients diagnosed after 2015 were excluded to ensure an adequate follow‐up time. And to select the accurate target group, we kept the sample of lung cancer patients with pathological diagnosis evidence and eliminated samples only diagnosed by less reliable diagnostic methods, such as clinical manifestation and radiography. Other exclusion criteria were as follows: (1) unknown TNM stage, unknown race, unknown metastases, and unknown treatment modality; (2) patients not diagnosed with positive histology or exfoliative cytology; and (3) patients diagnosed with death certificates only. For patients whose survival time was less than 1 month, the SEER database records the survival time as 0 months. Nevertheless, considering the accuracy of the data, the survival time of these patients was recorded as 0.5 months. The patient selection procedure is provided in Figure 1.

### 2.2 Comparison of the Cause of Death (COD) in Two Groups

To identify the difference in the COD between different age groups, the total cohorts were divided into the old group (aged > 45) and the young group (aged 18–45). All‐cause mortality and cancer‐specific mortality were analyzed and compared between the two groups. OS was defined as the time from diagnosis to all‐cause death while CSS was defined as the time from diagnosis to cancer‐specific death (CSD). Censored data was defined as patients alive at the time of analysis or lost to follow‐up. In addition, we counted competing mortality risk events in each group. The endpoint events consisted of CSD, other‐cause death (OCD), and censored. CSD was the primary endpoint of interest, and OCD was the competing event for CSD.

### 2.3 Predictive Variables and Construction of the Competing Risk Model

To predict the CSD and OCD of young advanced lung cancer patients, young patients had a random split into a training set and a validation set with a 7:3 ratio. The training set was used to establish the competing risk model, and the validation set was applied for external validation. Variables involved in the research included sex, age, race, TNM stage, bone metastasis, brain metastasis, lung metastasis, surgery, and chemotherapy. The distributional differences of each variable between the two cohorts were evaluated with the chi‐square test. Through univariate analysis, the probability of CSD and OCD in groups with different variables was calculated by applying the cumulative incidence function (CIF). Differences between groups were then assessed using Fine–Gray's test. Subsequently, the variables with *p* < 0.05 in the univariate competing risk analysis were incorporated into the proportional subdistribution hazard model as multivariate analysis. Based on the model, we obtained the subdistribution hazard ratio (sdHR) and 95% confidence interval (95% CI) of each variable. Eventually, the nomogram of the 1‐year, 3‐year, and 5‐year CSS rates was constructed based on the optimal regression model with significant predictive variables in the multivariate analysis (*p <* 0.05).

### 2.4 Validation of the Competing Risk Model

To evaluate the model performance, the area under the receiver operating characteristic curve (AUC), consistency index (C‐index), and calibration curve were applied to evaluate the discrimination and consistency of the competing risk model. The AUC was used to quantify the discrimination performance of the model, and a value greater than 0.7 indicates good discrimination [22]. The C‐index is a powerful indicator to evaluate the discrimination degree between the predicted value from the model and reality. The C‐index value ranged from 0.50 to 1.00. It is generally believed that a higher C‐index implied a better discrimination ability of the model [23]. In our study, the C‐index was calculated using the original dataset (1585 samples), and the 95% CIs were calculated through 1000 bootstrap samples. The calibration curve depicted the agreement between nomogram‐predicted probabilities and observed risks. The calibration curve falling on a 45° diagonal line implied the high prediction accuracy of a model [24].

### 2.5 Statistical Analysis

R software (version 3.4.1; http://www.r‐project.org) was used to perform all statistical analyses. The R packages cmprsk, rms, and mstate were utilized to develop and validate the competing risk model. All statistical tests were two‐sided, and *p* < 0.05 was considered to be statistically significant.

## 3 Results

### 3.1 Characteristics of Patients and Comparison of the COD in Two Groups

**FIGURE 2:**
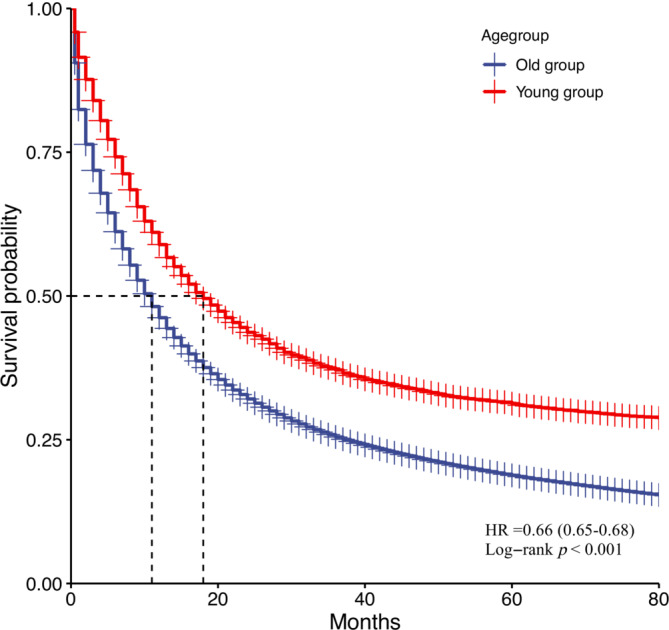
Cumulative Kaplan–Meier estimates of rates of all‐cause mortality for young lung cancer patients versus old lung cancer patients.

**FIGURE 3:**
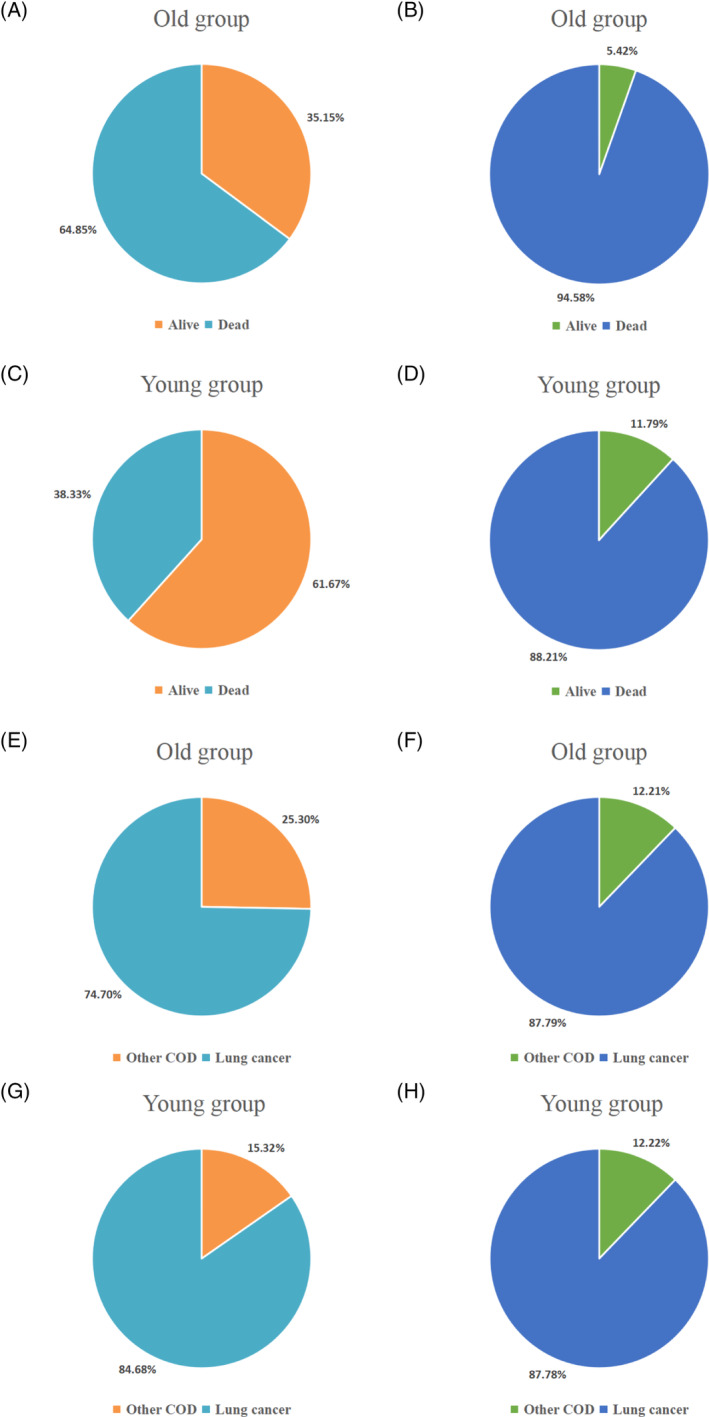
The causes of death analysis of young lung cancer patients and old lung cancer patients. (A, B) The proportion of the dead and surviving patients in the old group with early‐stage and advanced lung cancer. (C, D) The proportion of the dead and surviving patients in the young group with early‐stage and advanced lung cancer. (E, F) Cancer‐specific death and other‐cause death rate in the old group with early‐stage and advanced lung cancer. (G, H) Cancer‐specific death and other‐cause death rate in the young group with early‐stage and advanced lung cancer.

**FIGURE 4:**
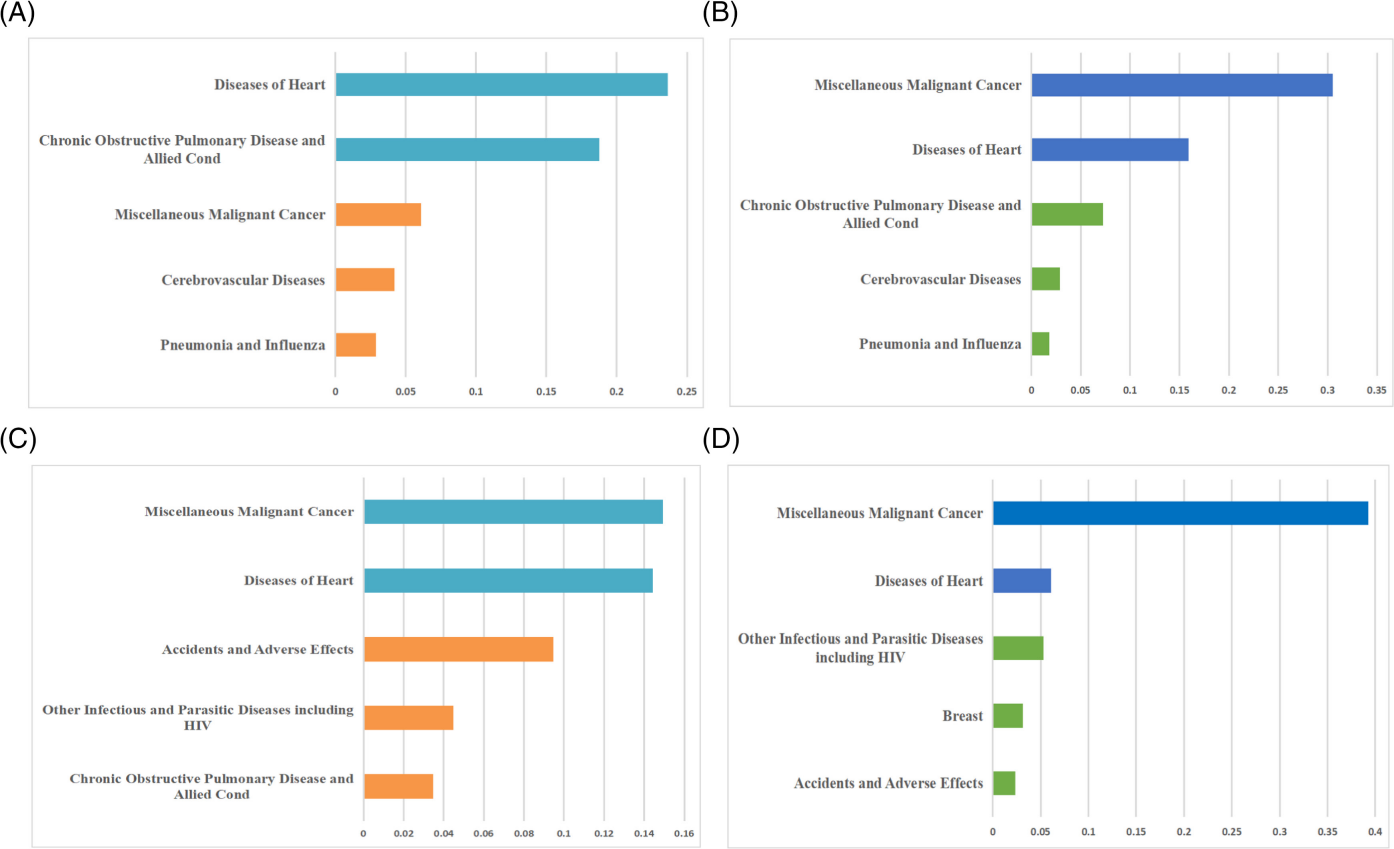
The categories of the causes of death of young lung cancer patients and old lung cancer patients. (A, B) The top‐5 main causes of death in the old group with early‐stage and advanced lung cancer. (C, D) The top‐5 main causes of death in the young group with early‐stage and advanced lung cancer.

A total of 322 207 old patients and 8147 young patients with advanced lung cancer were enrolled in our study. Compared with the old group, the prognosis of the young group was significantly better, with a median OS of 18 months in the young group and 11 months in the old group (HR = 0.66 (0.65–0.68), *p* < 0.001) (Figure 2). The death rate and COD of the two groups were compared further. The proportion of deaths in the old group was higher than that in the young group in both early and advanced stages of lung cancer (64.85% vs. 38.33%, *p* < 0.001, 94.58% vs. 88.21%, *p* < 0.001) (Figure 3A–D). In terms of the COD, the proportion of CSD in young patients was higher than in old patients with early‐stage lung cancer (84.68% vs. 74.70%, *p* < 0.001) while there is no difference in the advanced stage (87.78% vs. 87.79%, *p* = 0.999) (Figure 3E–H). Additionally, the categories and proportions of OCD varied from different age groups and different stages (Figure 4). Besides lung cancer, old patients with early‐stage lung cancer mainly died of some chronic diseases, such as heart disease and chronic obstructive pulmonary disease. In advanced‐stage lung cancer, death resulting from miscellaneous malignant cancer accounted for the largest proportion of OCD (Figure 4A,B). For young patients, miscellaneous malignant cancer and diseases of the heart were the common COD in both early and advanced stages (Figure 4C,D).

### 3.2 Factors Associated with CSS

**FIGURE 5:**
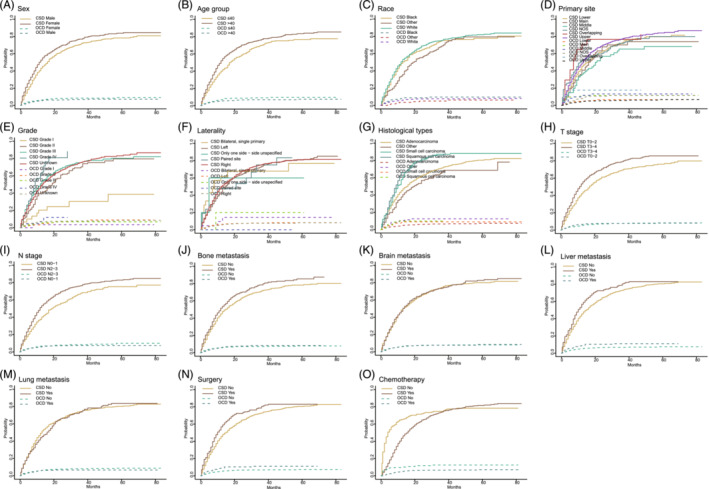
Cumulative incidence curve of cancer‐specific mortality and other‐cause death solid line: lung cancer‐specific death; dotted line: other‐cause death.

A total of 1585 young patients were randomly allocated into the training cohort (*n* = 1109) and validation cohort (*n* = 476) at a ratio of 7:3. The baseline characteristics of the two cohorts are shown in Table 1. Specifically, most of the patients in our study were female (50.5%), had lung adenocarcinoma (69%), and received chemotherapy therapy (80%). Through the univariate analysis, we calculated the 3‐year and 5‐year cumulative incidence of CSD and OCD grouped by different variables (Table 2 and Table S1). The corresponding CIF curves are presented in Figure 5. Based on Fine–Gray's test results, all factors, except primary site, laterality, brain metastasis, and lung metastasis, had strong correlations with CSD (*p* < 0.05). Patients with a high cumulative incidence of CSD were those with characteristics of male and older age. Earlier T and N stage, grade I, nonbone metastasis, and nonliver metastasis decreased the cumulative incidence of CSD. CIF for CSD differed significantly between those with and without surgery and chemotherapy. However, there existed few significant variables for the OCD event in the univariate analysis (Table S1) Through multivariate analysis, sex, age group, race, grade, histological types, T stage, N stage, bone metastasis, surgery, and chemotherapy were considered independent prognostic factors for CSS (*p* < 0.05) (Table 3).

**TABLE 1:** Clinical characteristics of young patients with advanced lung cancer.

Characteristics	Overall (*N* = 1585)	Training group (*N* = 1109)	Validation group (*N* = 476)	*p* value
Sex, *n* (%)				0.137
Female	801 (50.5%)	574 (36.2%)	227 (14.3%)	
Male	784 (49.5%)	535 (33.8%)	249 (15.7%)	
Age group, *n* (%)				0.911
≤ 40	566 (35.7%)	397 (25%)	169 (10.7%)	
> 40	1019 (64.3%)	712 (44.9%)	307 (19.4%)	
Race, *n* (%)				0.801
Other	239 (15.1%)	165 (10.4%)	74 (4.7%)	
White	1106 (69.8%)	772 (48.7%)	334 (21.1%)	
Black	240 (15.1%)	172 (10.9%)	68 (4.3%)	
Primary site, *n* (%)				0.697
Upper	761 (48%)	531 (33.5%)	230 (14.5%)	
Lower	393 (24.8%)	285 (18%)	108 (6.8%)	
Middle	90 (5.7%)	61 (3.8%)	29 (1.8%)	
NOS	219 (13.8%)	153 (9.7%)	66 (4.2%)	
Main	96 (6.1%)	62 (3.9%)	34 (2.1%)	
Overlapping	26 (1.6%)	17 (1.1%)	9 (0.6%)	
Grade, *n* (%)				0.230
Grade I	46 (2.9%)	30 (1.9%)	16 (1%)	
Grade II	188 (11.9%)	122 (7.7%)	66 (4.2%)	
Grade III	368 (23.2%)	271 (17.1%)	97 (6.1%)	
Grade IV	57 (3.6%)	42 (2.6%)	15 (0.9%)	
Unknown	926 (58.4%)	644 (40.6%)	282 (17.8%)	
Laterality, *n* (%)				0.903
Right—origin of primary	909 (57.4%)	632 (39.9%)	277 (17.5%)	
Left—origin of primary	608 (38.4%)	430 (27.1%)	178 (11.2%)	
Paired site, but no information concerning laterality	32 (2%)	21 (1.3%)	11 (0.7%)	
Bilateral, single primary	30 (1.9%)	21 (1.3%)	9 (0.6%)	
Only one side—side unspecified	6 (0.4%)	5 (0.3%)	1 (0.1%)	
Histological types, *n* (%)				0.966
Adenocarcinoma	1093 (69%)	764 (48.2%)	329 (20.8%)	
Squamous cell carcinoma	148 (9.3%)	103 (6.5%)	45 (2.8%)	
Other	155 (9.8%)	107 (6.8%)	48 (3%)	
Small cell carcinoma	189 (11.9%)	135 (8.5%)	54 (3.4%)	
T, *n* (%)				0.737
T0–T2	769 (48.5%)	535 (33.8%)	234 (14.8%)	
T3–T4	816 (51.5%)	574 (36.2%)	242 (15.3%)	
N, *n* (%)				0.598
N0–N1	399 (25.2%)	275 (17.4%)	124 (7.8%)	
N2–N3	1186 (74.8%)	834 (52.6%)	352 (22.2%)	
Bone metastasis, *n* (%)				0.400
Yes	674 (42.5%)	464 (29.3%)	210 (13.2%)	
No	911 (57.5%)	645 (40.7%)	266 (16.8%)	
Brain metastasis, *n* (%)				0.628
Yes	597 (37.7%)	422 (26.6%)	175 (11%)	
No	988 (62.3%)	687 (43.3%)	301 (19%)	
Liver metastasis, *n* (%)				0.326
Yes	332 (20.9%)	225 (14.2%)	107 (6.8%)	
No	1253 (79.1%)	884 (55.8%)	369 (23.3%)	
Lung metastasis, *n* (%)				0.099
Yes	438 (27.6%)	293 (18.5%)	145 (9.1%)	
No	1147 (72.4%)	816 (51.5%)	331 (20.9%)	
Surgery, *n* (%)				0.588
No	1463 (92.3%)	1021 (64.4%)	442 (27.9%)	
Yes	122 (7.7%)	88 (5.6%)	34 (2.1%)	
Chemotherapy, *n* (%)				0.603
Yes	1268 (80%)	891 (56.2%)	377 (23.8%)	
No	317 (20%)	218 (13.8%)	99 (6.2%)	

**TABLE 2:** Univariate competing risk analysis for lung cancer‐specific mortality.

Characteristic	*N* (%)	Cause‐specific death		
		3 years	5 years	*p* value
Sex				< 0.05
Female	574 (36.2%)	0.71855768	0.78288588	
Male	535 (33.8%)	0.76948964	0.83754085	
Age group				< 0.001
≤ 40	397 (25%)	0.69957152	0.76533084	
> 40	712 (44.9%)	0.76533084	0.83391483	
Race				< 0.05
Other	165 (10.4%)	0.68836847	0.79272866	
White	772 (48.7%)	0.75285776	0.81890457	
Black	172 (10.9%)	0.75162254	0.77744679	
Primary site				0.07
Upper	531 (33.5%)	0.77303671	0.83986175	
Lower	285 (18%)	0.72976409	0.79602069	
Middle	61 (3.8%)	0.65272420	0.68346191	
NOS	153 (9.7%)	0.71893455	0.79844682	
Main	62 (3.9%)	0.70107527	0.73820455	
Overlapping	17 (1.1%)	0.76470588	NA	
Grade				< 0.001
Grade I	30 (1.9%)	0.30404858	0.38671559	
Grade II	122 (7.7%)	0.70390142	0.79572840	
Grade III	271 (17.1%)	0.75628713	0.80334633	
Grade IV	42 (2.6%)	NA	NA	
Unknown	644 (40.6%)	0.75678101	0.83183073	
Laterality				0.95
Right—origin of primary	632 (39.9%)	0.75328798	0.80882804	
Left—origin of primary	430 (27.1%)	0.73290633	0.81200617	
Paired site, but no information concerning laterality	21 (1.3%)	0.75056689	NA	
Bilateral, single primary	21 (1.3%)	0.67857143	0.76785714	
Only one side—side unspecified	5 (0.3%)	0.60000000	0.60000000	
Histological types				< 0.001
Adenocarcinoma	764 (48.2%)	0.71473405	0.80309137	
Squamous cell carcinoma	103 (6.5%)	0.85498729	NA	
Other	107 (6.8%)	0.66771318	0.68665840	
Small cell carcinoma	135 (8.5%)	0.87148943	0.88131068	
T				< 0.001
T0–T2	535 (33.8%)	0.69324297	0.76089653	
T3–T4	574 (36.2%)	0.79144800	0.85681538	
N				< 0.001
N0–N1	275 (17.4%)	0.67795561	0.75230586	
N2–N3	834 (52.6%)	0.76450015	0.82857325	
Bone metastasis				< 0.05
Yes	464 (29.3%)	0.78415510	0.85376532	
No	645 (40.7%)	0.71505124	0.78185209	
Brain metastasis				0.71
Yes	422 (26.6%)	0.73620620	0.82664110	
No	687 (43.3%)	0.74718216	0.79959611	
Liver metastasis				< 0.05
Yes	225 (14.2%)	0.80325544	0.82921987	
No	884 (55.8%)	0.72883218	0.80367857	
Lung metastasis				0.59
Yes	293 (18.5%)	0.73980860	0.83654934	
No	816 (51.5%)	0.74478066	0.80148063	
Surgery				< 0.001
Yes	88 (5.6%)	0.44805986	0.49907683	
No	1021 (64.4%)	0.76902585	0.83909732	
Chemotherapy				< 0.001
Yes	891 (56.2%)	0.74175276	0.81568290	
No	218 (13.8%)	0.74713443	0.78366941	

**TABLE 3:** Proportional subdistribution hazard model for lung cancer‐specific mortality.

	Coefficient	SHR	95% CI	*p* value
Sex (male vs. female)	0.1665	1.1811104	1.05–1.33	0.0217
Race				
Other	−0.3214	0.7251344	0.58–0.91	0.0187
White	−0.041	0.9598195	0.81–1.14	0.6894
Age group (> 40 vs. ≤ 40)	0.344	1.4105482	1.24–1.6	< 0.0001
Grade				
Grade I	—	—	—	—
Grade II	0.8293	2.2918007	1.28–4.11	0.0193
Grade III	1.0089	2.7425402	1.55–4.84	0.0035
Grade IV	1.113	3.043482	1.61–5.74	0.0039
Unknown	0.8949	2.4471182	1.39–4.31	0.0092
Histological types				
Adenocarcinoma	—	—	—	—
Other	−0.1839	0.8319993	0.67–1.03	0.1651
Small cell carcinoma	0.2349	1.2648266	1.05–1.53	0.0393
Squamous cell carcinoma	0.3103	1.3638382	1.12–1.67	0.011
T Stage (T3–T4 vs. T1–T2)	0.2915	1.3384993	1.18–1.51	< 0.0001
N Stage (N2–N3 vs. N1)	0.2085	1.2318777	1.07–1.42	0.0173
Surgery (yes vs. no)	−0.9171	0.399679	0.3–0.53	< 0.0001
Chemotherapy (yes vs. no)	−0.6803	0.5064815	0.44–0.59	< 0.0001
Bone metastasis (yes vs. no)	0.1897	1.2088841	1.07–1.37	0.0117
Liver metastasis (yes vs. no)	0.1204	1.1279144	0.97–1.31	0.183

### 3.3 Nomogram Construction and Validation Based on Competing Risk Model

**FIGURE 6:**
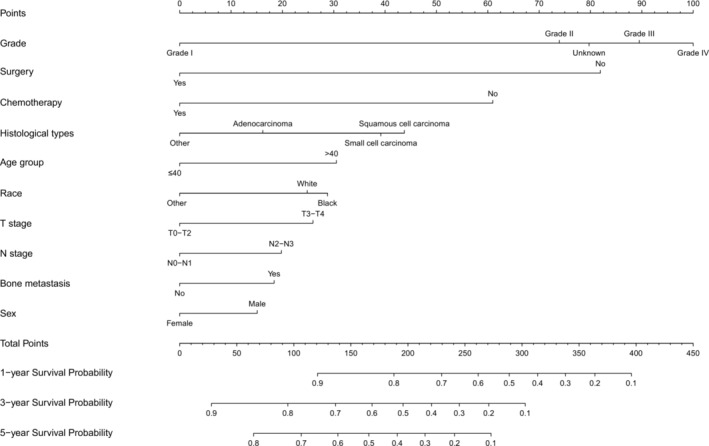
Nomogram for predicting cancer‐specific survival in young patients with advanced lung cancer.

**FIGURE 7:**
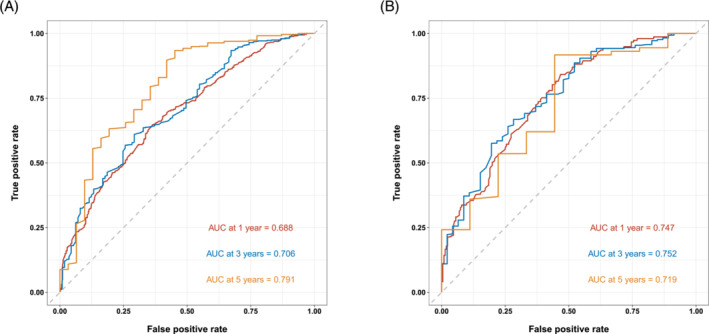
ROC curve for predicting the cancer‐specific survival in young patients with advanced lung cancer. (A) ROC curves of 1‐, 3‐, and 5‐year CSS rates in the training set. (B) ROC curves of 1‐, 3‐, and 5‐year CSS rates in the validation set.

**FIGURE 8:**
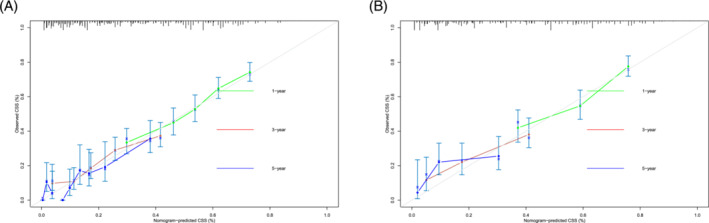
Calibration curves of 1‐, 3‐, and 5‐year survival for predicting the young patients with advanced lung cancer in the training set and the validation set.

Based on the independent prognostic factors, a nomogram was constructed to predict the probability of CSS for young patients with advanced lung cancer (Figure 6). Grade made the largest contribution to the prognosis of young patients, followed by surgery, chemotherapy, and histological types. The total score was the sum of the score of each factor, and the 1‐, 3‐, and 5‐year survival rates were calculated by drawing a vertical line from the total score on the nomogram. The AUC for the competing risk nomogram in the 1‐, 3‐, and 5‐year prediction of CSD was 0.688, 706, and 791 and 0.747, 0.752, and 0.719 in the training cohort and the validation cohort, respectively (Figure 7). The predicted calibration curves were close to the standard curves for 1‐, 3‐, and 5‐year survival in both the training set and validation set, which revealed high consistency of the nomogram (Figure 8). Through the cross‐validation by bootstrap, the C‐index values were 0.692 (95% CI: 0.636–0.747), indicating that the predictive model had great discrimination.

## 4 Discussion

Our retrospective study indicates that young lung cancer is a distinct entity from elderly patients, with significantly different competitive risk events. Furthermore, to the best of our knowledge, this is the first study to establish a competitive risk nomogram model for advanced young lung cancer.

The current results regarding the prognosis of young lung cancer patients remain controversial. Thomas et al. discovered that both OS and lung CSS (LCSS) of young patients were superior to those of elderly patients [7]. However, some research reported that the prognosis had no significant difference between metastatic young patients and older patients [11]. In our study, by comparing COD analysis, we also indicate that the median CSS and OS of young lung cancer patients are significantly better than those of elderly patients. However, the positive effect of age on prognosis shrinks with disease progression. For early‐stage lung cancer, age is a highly significant prognosis protective factor. Arnold et al. conducted a study to stratify patients with NSCLC into young and middle‐aged/elderly groups and then found that among stage I and II patients, the median OS and 5‐year survival rates of young patients were superior to those of middle‐aged/elderly patients [25]. And the difference has narrowed significantly with the progression of cancer staging [26], which may contribute that disease itself plays a dominant role in determining the patient prognosis in advanced stages of lung cancer. Whereas in the early stages of the disease, the prognosis of elderly patients is affected by both the disease itself and comorbidities associated with the age [11].

For lung cancer patients, the impact of comorbidity on prognosis will gradually increase with the extension of survival time [27]. Therefore, analyzing the COD in young patients with advanced lung cancer may facilitate the development of oncologic treatments and care strategies, which can improve the prognostic outcomes ultimately. In our study, we found that the major competing risk events of advanced young lung cancer were mostly consistent with those of old patients, including other malignancies and heart‐related diseases. Nevertheless, compared to older patients, young patients with advanced lung cancer have a lower proportion of deaths attributed to comorbidities such as cardiovascular disease, COPD, and cerebrovascular disease, which are commonly associated with smoking in the elderly population [28]. It is worthwhile to note that young lung cancer patients have a higher proportion of nontumor deaths from cardiovascular, which may be relevant to the toxicity of cancer treatment, including radiotherapy and chemotherapy [29]. Additionally, we have noticed a higher proportion of accidents and adverse effects in young patients with lung cancer. These findings underscore the need for more attention to adverse event management in the treatment of young patients with lung cancer, as well as the importance of addressing their psychological well‐being.

Previous studies on the prognosis of advanced‐stage young lung cancer have mainly relied on OS, with little consideration for the impact of competing events. And non–lung cancer‐specific mortality may interfere with the prediction of lung cancer‐specific mortality when there are competing events for the outcome [30, 31]. Therefore, it is more appropriate to apply the competing risk model to construct the prognostic model in the presence of competing events. Peng and Sun established a nomogram for OS and CSS for young NSCLC, both of which outperformed the TNM staging system in terms of predictive performance [21]. Despite establishing a nomogram based on the competitive risk model, their study was restricted to NSCLC and failed to take into account risk factors such as metastasis. Considering the urgent need for clinical prognostic guidance in late‐stage young adult lung cancer, we have developed and validated a nomogram based on a large population from the SEER database.

In the present study, 10 independent risk factors associated with CSS, which involve sex, age group, race, grade, histological types, T stage, N stage, bone metastasis, surgery, and chemotherapy were identified through competing risk analysis for young patients with advanced lung cancer. Consistent with previous studies [25, 32], our research found that advanced‐stage young lung cancer patients were predominantly adenocarcinoma, females, which were both protective prognostic factors for lung cancer patients. These patients also tend to have more concomitant mutated genes [3]. Multiple studies have shown that the genetic features of young‐onset lung cancer patients are distinct from those of older patients, which makes young‐onset lung cancer patients benefit from targeted therapies directed at these genes [33].

After integrating all independent prognostic factors, we developed a nomogram based on a competitive risk model to predict tumor‐specific survival. Our model demonstrated high discriminative and predictive accuracy as evidenced by high C‐index and AUC values. The calibration curves showed excellent consistency between the predicted and observed probabilities of 1‐year, 2‐year, and 5‐year CSS, both in the training and validation sets. In clinical applications, our model is user‐friendly as all parameters are readily accessible.

Nonetheless, it is necessary for us to further investigate the parameters in our model. We analyzed multiple types of metastases in contrast to Peng's study and subsequently incorporated bone metastasis into our model. Previous studies have shown that young NSCLC patients are at a higher risk of bone metastasis, which is probably attributed to higher bone marrow flow and circulating tumor cells in the skeletal system of young patients [34, 35]. All of these indicate that bone metastasis is a crucial indicator in the prognosis of young adult lung cancer when evaluating prognosis. Furthermore, in our model, there is a higher proportion of pathological staging, surgery, and chemotherapy. Especially, the risk scores of no surgery and no chemotherapy were both higher than 60 points, indicating a significant impact on prognosis and playing a momentous role in guiding clinical practice. The conventional perspective holds that the treatment of metastatic lung cancer is mainly palliative rather than surgical [36]. Recent studies have indicated that surgery may be appropriate for particular patients with advanced NSCLC who have limited metastases and can bring significant survival benefits [37, 38]. Additionally, studies have shown that younger age, smaller primary lung tumors, N0 stage, and receiving lobectomy have better surgical benefits in advanced patients [37]. As mentioned above, young patients have fewer comorbidities and better treatment tolerance. Combined with our model, we believe that young patients with limited metastasis in advanced lung cancer may benefit from surgery.

We acknowledge certain limitations in our study. First, this study was limited by the data collection of a retrospective study, leading to unavoidable bias. Additionally, although we found some potential benefits from active therapy for young patients with advanced lung cancer, SEER database does not include detailed treatment information, thereby affecting the further adoption of this finding to the clinical application. Future studies will need to collect the concrete surgery types and chemotherapeutic agents in the prospective study to identify the specific subgroup of young lung cancer benefiting from the active therapy. Secondly, previous studies have shown that the genetic characteristics of young lung cancer patients different from the elderly patients are related to their better treatment prognosis [33]. Since the genetic data were not addressed in the SEER database, our study lacks further exploration of the relationship between genes and prognosis. For future research, studying the genetic characteristics of specific young lung cancer patients who benefit from positive treatment can be conducive to guiding the clinical decision of treatment plans.

## 5 Conclusion

In our manuscript, we found that young lung cancer is a distinct entity from elderly patients with significantly different prognoses and the spectrum of competing risk events. Our study also proposed a useful tool to predict the CSS of young patients with advanced lung cancer. Our finding may facilitate personalized clinical decisions, suggesting beware of the adverse effects of the treatment process on the heart of the patients and indicating potential benefits from the surgery for a subgroup of young patients with advanced lung cancer.

## Author Contributions

Conception and design: Jiaxin Li, Bolin Pan, Liqiang Wang, Chengzhi Zhou, and Ming Liu. Provision of study materials: Qiying Huang, Honglang Zhang, Xiaohong Xie, Xinqin Lin, Ming Liu, and Liqiang Wang. Data analysis and interpretation: Jiaxin Li, Chulan Zhan, Tong Lin, and Yangzhi Qiu. Manuscript writing: Jiaxin Li, Bolin Pan, and Liqiang Wang. Final approval of the manuscript: all authors.

## Ethics Statement

The authors are accountable for all aspects of the work in ensuring that questions related to the accuracy or integrity of any part of the work are appropriately investigated and resolved. The data released by the SEER database do not require informed patient consent because cancer is a reportable disease in every state of the United States.

## Conflicts of Interest

The authors declare no conflicts of interest.

## Supporting information


**Table S1** Univariate competing risk analysis for other‐cause death.

## Data Availability

The data that support the findings of this study are available from the corresponding author upon reasonable request.
