# Supplementary material for: A Nomogram for Predicting Cancer‐Specific Survival in Young Patients With Advanced Lung Cancer Based on Competing Risk Model
Source: Clin Respir J. 2024 Aug 7;18(8):e13800. doi: 10.1111/crj.13800 (PMC11306286; doi:10.1111/crj.13800)
Supplement: Supplementary file 1 — Table S1 Univariate competing risk analysis for other‐cause death. [file CRJ-18-e13800-s001.docx]

| **Supplementary Table 1 Univariate competing risk analysis for other-cause death** | | | | |
| --- | --- | --- | --- | --- |
| **Characteristic** | **Other-cause death** | | | |
|  | **3-year** | **5-year** | ***p* value** |  |
| **Sex** |  |  | 0.32 |  |
| Female | 0.08466275 | 0.09096996 |  |  |
| Male | 0.06758239 | 0.07149738 |  |  |
| **Age group** |  |  | 0.16 |  |
| ≤40 | 0.09080225 | 0.09547327 |  |  |
| >40 | 0.06841375 | 0.07415912 |  |  |
| **Race** |  |  | 0.37 |  |
| Black | 0.09680034 | 0.09680034 |  |  |
| Other | 0.05101312 | 0.06285941 |  |  |
| White | 0.07729391 | 0.08180702 |  |  |
| **Primary Site** |  |  | 0.03 |  |
| Lower | 0.06561891 | 0.06561891 |  |  |
| Main | 0.11327832 | 0.11327832 |  |  |
| Middle | 0.13211186 | 0.13211186 |  |  |
| NOS | 0.11467147 | 0.11467147 |  |  |
| Overlapping | 0.17647059 | NA |  |  |
| Upper | 0.05701204 | 0.06665009 |  |  |
| **Grade** |  |  | 0.67 |  |
| Grade I | 0.03461538 | 0.03461538 |  |  |
| Grade II | 0.07727685 | 0.09030195 |  |  |
| Grade III | 0.06837208 | 0.06837208 |  |  |
| Grade IV | NA | NA |  |  |
| Unknown | 0.07817119 | 0.08509412 |  |  |
| **Laterality** |  |  | 0.41 |  |
| Bilateral, single primary | 0.14285714 | 0.14285714 |  |  |
| Left | 0.07725681 | 0.08189568 |  |  |
| Only one side - side unspecified | 0.20000000 | 0.20000000 |  |  |
| Paired site, but no information concerning laterality | 0.00000000 | NA |  |  |
| Right | 0.07523689 | 0.08075761 |  |  |
| **Histological types** |  |  | 0.12 |  |
| Adenocarcinoma | 0.06475890 | 0.07240462 |  |  |
| Other | 0.12388934 | 0.12388934 |  |  |
| Small cell carcinoma | 0.08922559 | 0.08922559 |  |  |
| Squamous cell carcinoma | 0.09804742 | NA |  |  |
| **T stage** |  |  | 0.85 |  |
| T0-T2 | 0.07631759 | 0.08282902 |  |  |
| T3-T4 | 0.07640506 | 0.07980590 |  |  |
| **N stage** |  |  | 0.43 |  |
| N0-N1 | 0.08319357 | 0.10003471 |  |  |
| N2-N3 | 0.07419793 | 0.07419793 |  |  |
| **Bone metastasis** |  |  | 0.67 |  |
| No | 0.07145880 | 0.07913372 |  |  |
| Yes | 0.08404277 | 0.08404277 |  |  |
| **Brain metastasis** |  |  | 0.96 |  |
| No | 0.07673400 | 0.07933196 |  |  |
| Yes | 0.07571181 | 0.08384907 |  |  |
| **Liver metastasis** |  |  | 0.05 |  |
| No | 0.06794161 | 0.07399143 |  |  |
| Yes | 0.11019646 | 0.11019646 |  |  |
| **Lung metastasis** |  |  | 0.44 |  |
| No | 0.08017717 | 0.08649484 |  |  |
| Yes | 0.06595905 | 0.06595905 |  |  |
| **Surgery** |  |  | 0.61 |  |
| No | 0.07484449 | 0.08061019 |  |  |
| Yes | 0.09578846 | 0.09578846 |  |  |
| **Chemotherapy** |  |  | <0.05 |  |
| No | 0.12499312 | 0.12499312 |  |  |
| Yes | 0.06478267 | 0.07106667 |  |  |
